# First International Conference on Lysophospholipids and Related Bioactive Lipids in Biology and Disease Sponsored by the Federation of American Societies of Experimental Biology

**DOI:** 10.1100/tsw.2001.65

**Published:** 2001-07-17

**Authors:** Edward J. Goetzl, Gabor J. Tigyi, Timothy Hla

**Affiliations:** Department of Medicine, University of California San Francisco, UB8B, 533 Parnassus at 4th, San Francisco, CA 94143-0711, USA

**Keywords:** development, differentiation, growth, cytoprotection, apoptosis, angiogenesis, carcinogenesis, neurodegeneration, neurotransmission

## Abstract

The First International Conference on “Lysophospholipids and Related Bioactive Lipids in Biology and Diseases” was held in Tucson, AZ on June 10–14, 2001, under the sponsorship of the Federation of American Societies of Experimental Biology (FASEB). More than 100 scientists from 11 countries discussed the recent results of basic and clinical research in the broad biology of this emerging field. Immense progress was reported in defining the biochemistry of generation and biology of cellular effects of the bioactive lysophospholipids (LPLs). These aspects of LPLs described at the conference parallel in many ways those of the eicosanoid mediators, such as prostaglandins and leukotrienes. As for eicosanoids, the LPLs termed lysophosphatidic acid (LPA) and sphingosine 1-phosphate (S1P) are produced enzymatically from phospholipid precursors in cell membranes and act on cells at nanomolar concentrations through subfamilies of receptors of the G protein–coupled superfamily. The rate-limiting steps in production of LPLs were reported to be controlled by specific phospholipases for LPA and sphingosine kinases for S1P. The receptor subfamilies formerly were designated endothelial differentiation gene-encoded receptors or Edg Rs for their original discovery in endothelial cells. A currently active nomenclature committee at this conference suggested the ligand-based names: S1P_1_ = Edg-1, S1P_2_ = Edg-5, S1P_3_ = Edg-3, S1P_4_ = Edg-6, and S1P_5_ = Edg-8; LPA_1_ = Edg-2, LPA_2_ = Edg-4, and LPA_3_ = Edg-7 receptors. Several families of lysophospholipid phosphatases (LPPs) have been characterized, which biodegrade LPA, whereas S1P is inactivated with similar rapidity by both a lyase and S1P phosphatases.

The protean roles of LPA, S1P, and related bioactive lipids were reported to encompass some of the effector activities of the eicosanoids and also to include mediation of cellular proliferation, differentiation, survival, and malignant transformation. LPA and S1P appear to be most distinctively active for blood cells, especially platelets, endothelial cells and vascular smooth muscle cells, cardiomyocytes, T lymphocytes and macrophages, preadipocytes, neurons and astrocytes, and in atherosclerosis and cancer. Genetic deletions of some of the LPA or S1P receptors (Edg Rs) in knockout mice were described for the first time at this conference. A few of these genetic disruptions have revealed profound and sometimes unexpected phenotypes, which elucidated important physiological contributions of these mediators. For example, the $S1P_1$ (Edg-1) knockout is embryonically lethal due to failure of the vascular smooth muscle cells to surround blood vessels completely and to establish vascular integrity, despite normal morphogenetic patterns of angiogenesis. The $LPA_1$ (Edg-2) knockout has subtle olfactory and neonatal behavioral abnormalities. Quite unexpectedly, the $S1P_2$ (Edg-5) knockout mouse has behavioral defects and cortical seizures. Mutations in the miles apart gene, which encodes a homolog of $S1P_2$ (Edg-5) in zebrafish, reportedly led to failure of cardiac development from lack of fusion of the two halves of the heart. Further, high expression of some LPA and S1P receptors was seen in cancer cells whereas they were absent from corresponding normal cells, such as the upregulation of $LPA_2$ (Edg-4) and $LPA_3$ (Edg-7) in ovarian cancer cells. In these instances, direct pathogenetic involvement of LPA and S1P remain to be proven. Disruption of genes encoding some lysophospholipid phosphatases (LPPs) were reported to have resulted in major derangements of *Drosophila* and murine development. In addition, pharmacological manipulation of sphingosine kinases and S1P phosphatases have delineated critical involvement of S1P and other sphingolipids in cellular survival and neoplasia.

The consensus of conferees was that the LPL systems offer abundant pharmacological targets which should permit meaningful manipulation of the tissue and fluid levels of these lipid mediators, as well as of the expression and functions of receptors for LPA and S1P. A small number of synthetic analogs of the index lipids reported at the conference are either agonists or antagonists of at least micromolar potency and sometimes distinctive specificity. However, almost all lack bioavailability sufficient for therapeutic efficacy. Many pharmaceutical firms now are pursuing discovery of inhibitors of enzymes critical in some of the LPA- and S1P-generating pathways, and LPA and S1P receptor agonists and antagonists suitable for treatment of several cardiovascular, neurological, and malignant diseases.

The Second International Conference on this topic is scheduled for July 2003. Suggested additional reading on the subject are:

- Goetzl, E.J. and Lynch, K.R. (2000) Lysophospholipids and eicosanoids in biology and pathophysiology. *Ann. N.Y. Acad. Sci*. 905.
- Hla, T. (2001) Glycero- and sphingo-lysolipid phosphates: biochemistry, cell biology, pharmacology and function of an emerging class of lipid mediators. *Prostaglandins and Other Lipid Mediators, Suppl.* Elsevier, New York.
- Goetzl, E.J. and Tigyi, G. (2001) Lysophospholipids and related lipid mediators in biology and diseases. *Biochem. Biophys. Acta*, in press.
